# Intergeneric Hybrid from *Jatropha curcas* L. and *Ricinus communis* L.: Characterization and Polyploid Induction

**DOI:** 10.3390/biology8020050

**Published:** 2019-06-22

**Authors:** Duangporn Premjet, Abraham Kusi Obeng, Anupan Kongbangkerd, Siripong Premjet

**Affiliations:** 1 Center of Excellent in Research for Agricultural Biotechnology, Faculty of Agriculture, Natural Resources and Environment, Naresuan University, Muang, Phitsanulok 65000, Thailand; duangpornp@nu.ac.th (D.P.); anupank@nu.ac.th (A.K.); 2 Department of Agricultural Science, Faculty of Agriculture, Natural Resources and Environment, Naresuan University, Muang, Phitsanulok 65000, Thailand; 3 Department of Biology, Faculty of Science, Naresuan University, Muang, Phitsanulok 65000, Thailand; aobeng@uds.edu.gh

**Keywords:** embryo rescue, gene introgression, *Jatropha curcas* L., polyploidization, RAPD marker, *Ricinus communis* L.

## Abstract

*Jatropha curcas* L. (2n = 2× = 22) is increasingly attracting attention in the biodiesel industry for its oil. However, the cultivation of *J. curcas* L. is faced with numerous challenges unlike the cultivation of *Ricinus communis* L. (2n = 2× = 20), a closely related species. The generation of an intergeneric hybrid between *J*. *curcas* L. and *R. communis* L. was investigated. Intergeneric hybrids were produced by hand crossing. Immature embryos were rescued, in vitro, from the hybrid seeds and cultured on an enriched Murashige and Skoog (MS) medium for a month. The plantlets produced were grown in sterile peat moss in plastic pots and covered with polyethylene for 30 days, after which they were transferred into cement potted soil. The hybridity and the genuineness of the hybrids were successfully confirmed using randomly amplified polymorphic DNA (RAPD) markers. The number of branches, stem diameter, and leaf size of the F_1_ hybrids were similar to those of *J*. *curcas* L. while the plant height was similar to that of *R. communis* L. Young hybrids were treated with various concentrations (0%, 0.3%, 0.4%, and 0.5%) of colchicine to induce polyploids. The calli (JR6) treated with 0.3% colchicine recorded the highest tetraploid cell percentage (38.89%). A high tetraploid cell percentage (>50%) is significant in overcoming the problem of sterility after hybridization.

## 1. Introduction

*Jatropha curcas* L. (2n = 2× = 22), a perennial shrub, and *Ricinus communis* L. (2n = 2× = 20), a tropical and sub-tropical perennial shrub or temperate annual, are two very promising non-edible oilseed plants in the family Euphorbiaceae [1,2]. *J*. *curcas* L. has generated a lot of interest as a sustainable alternative source of seed oil for the production of biodiesel. On the other hand, *R*. *communis* L. is a source of medicinal and vegetable oil with numerous industrial and medical uses worldwide [3].

In spite of its potential in the biodiesel industry, commercial production of *J*. *curcas* L. is faced with numerous challenges including poor seed yield and non-synchronous flowering. Seed cultivation of *J*. *curcas* L. is often associated with heterozygosity, which affects the quality and quantity of the oil content. Propagation by cuttings also yields plants that are susceptible to drought and diseases [4]. Attempts to improve *J*. *curcas* L. through conventional breeding, radiation mutation, protoplast fusion and/or in vitro induction of polyploids has failed to produce improved varieties with the required characteristics [5,6]. However, the genetic modification and manipulation of *J*. *curcas* L., by incorporating genes that code for novel traits from closely related species to generate an intergeneric F_1_ hybrid, offer the opportunity for improvement.

Crop development research worldwide is mainly focused on the improvement of the genetic makeup through techniques such as interspecific hybridization and genetic transformation via *Agrobacterium tumefaciens* and microprojectile bombardment [3,7]. Research into the genetic diversity of closely related crops helps in understanding their genetic makeup for improvement. The genetic makeup of *Jatropha*-related species such as *R*. *communis* L. has been extensively studied and reported [1,8]. *R*. *communis* L. possesses beneficial genetic traits for improving *J*. *curcas*. It is a wild plant that has been developed into an herbaceous crop with synchronous seeds, giving it a high seed yield that is easily harvested [9]. The development of an intergeneric F_1_ hybrid between *J*. *curcas* L. and *R*. *communis* L. is very important in improving *Jatropha*. Sexual crossing between *J*. *curcas* L. and *R*. *communis* L. may facilitate introgression of traits such as synchronous seed, high seed yield, and the herbaceous nature of *R*. *communis* L. to *J*. *curcas* L. However, pre- and post-fertilization barriers affect the hybridization process [10]. Laosatit et al. [10] reported on embryo abortion, a post-fertilization barrier, which was overcome by ovule culture. Sterility of F_1_ hybrids is also very common in intergeneric hybridization [11]. However, the fertility of F_1_ hybrids can be restored by doubling their genome through polyploidization [12]. Polyploidy manipulation is a very important technique to genetically improve plants. Polyploids can be induced in the F_1_ hybrids by the application of antimitotic agents. Traditionally, colchicine has been successfully used to induce polyploids [6].

Molecular markers play a very significant role in the identification and confirmation of F_1_ hybrids during plant breeding [13]. A number of molecular marker techniques have been established, among which, the randomly amplified polymorphic DNA (RAPD) has been extensively studied and successfully used in plant breeding [14]. The RAPD analysis has been widely used for studying genetic relationships among closely related species [15]. Developing a technique to create intergeneric F_1_ hybrids between *J*. *curcas* L. and *R*. *communis* L. was therefore investigated in this study.

## 2. Materials and Methods

### 2.1. Plant Materials

Accessions of *J*. *curcas* L. cultivar “No. 5”, a local species at the agricultural experiment field, Faculty of Agriculture, Natural Resources, and Environment, Naresuan University, Thailand and *R*. *communis* L. cultivar “TCO208”, from Thai Castor Oil Industries Co., Ltd., Bangkok, Thailand, were used in the current study. *J*. *curcas* L. cultivar “No. 5” is a nontoxic species whilst *R*. *communis* L. cultivar “TCO208” is a well-developed and best species for castor oil production in Thailand. The two cultivars were grown on the field at the Agricultural Science farm, Faculty of Agriculture, Natural Resources and Environment, Naresuan University, Phitsanulok, Thailand.

### 2.2. Intergeneric Hybridization

Sexual intergeneric crosses and reciprocal crosses were performed between the female parent, *J*. *curcas* L., and the male parent, *R*. *communis* L. in 2012 and the F_1_ hybrids were maintained until 2017. Emasculation of the male flower was carried out following the method described by Rehman and colleagues [16]. Emasculation was undertaken two to three days before anthesis to avoid self-pollination. The emasculated inflorescence was covered with a paper bag for two days. After flowering, the stigma of individual female plants was sprayed with different concentrations of gibberellic acid (GA_3_), 1-naphthaleneacetic acid (NAA), and calcium–boron formulation (Table 1) two days before and two days after pollination. Artificial pollination by hand was carried out between 8.00 and 10.00 a.m. for seven days, after which the pollinated plants were protected with paper bags. The percentage of fruit set, seed set, and embryo development were observed for up to 40 days after pollination.

### 2.3. Embryo Rescue of the Intergeneric F_1_ Hybrids

Intergeneric F_1_ hybrid fruits were harvested 40 days after pollination. The seeds were then excised and the surface immersed in ethanol (70%) for 30 s, followed by sterilization in 10% Clorox for 10 min and finally rinsed in sterile distilled water three times. Young embryos were removed from the seeds and cultured on a regeneration medium (RM). The composition of the RM (pH 5.7) included Murashige and Skoog (MS) medium [17] enriched with citric acid (30 mg/L), 6-benzylaminopurine (BA, 1 mg/L), indole-3-butyric acid (IBA, 0.25 mg/L), polyvinylpyrrolidone (PVP, 500 mg/L), sucrose (3% *w/v*), kinetin (Kn, 0.5 mg/L), and agar (0.7% *w/v*) [18]. The cultured embryos were incubated for four weeks at 25 °C under a 16 h light and 8 h dark photoperiod. After four weeks, the plantlets were grown in sterile peat moss in small plastic pots and covered with polyethylene bags for 30 days. The young seedlings that developed after 30 days were transferred into cement potted soil in the field.

### 2.4. Morphological Characterization

The morphological characteristics of both the parental and intergeneric F_1_ hybrid plants were evaluated according to [19,20]. The evaluated characteristics were stem diameter (mm), plant height (mm), total number of branches per plant, number of female and male flowers, seed size (length and width, mm), and leaf size (length and width, mm).

### 2.5. DNA Isolation

Genomic DNA was extracted from fresh young leaves and purified using Omega Plant DNA Kit (D3485-02, Omega Bio-tek, Norcross, GA, USA) according to the manufacturer’s protocol. The extracted DNA was quantified using the UV spectrophotometer (Analytik Jena Specord 40, Analytik Jena AG, Jena, Germany) at the absorbance ratio of 260/280 nm. The DNA concentration was adjusted to 100 ng/mL.

### 2.6. Randomly Amplified Polymorphic DNA (RAPD) Marker Analysis

A total of 46 random primers (Operon Technology Inc., Alameda, CA, USA) were screened against the template DNA. A reaction volume of 25 µL containing template DNA (100 ng), 1× PCR buffer (with 15 mM Mg^2+^), 2 mM MgCl_2_, 0.2 mM dNTP mix (Fermentus Pvt. Ltd., Bangalore, India), single primer (0.8 µM), and Taq DNA polymerase (0.5 U) was used. For the initial denaturation, the thermocycler (GeneAmp PCR System 9700, Applied Biosystems, Foster City, CA, USA) was set at 94 °C for 3 min, followed by 45 cycles consisting of 1 min denaturing at 94 °C, annealing for 1 min at 37 °C, 2 min of extension at 72 °C and finally, 7 min of extension at 72 °C. The amplification products (10 µL) were electrophoresed through 1.5% (w/v) agarose gel (Research Organics Inc., Cleveland, OH, USA) in 1× Tris-acetate-EDTA (TAE) at 100 V for 30 min. This was followed by staining with 0.5 µg/mL ethidium bromide. The resulting fragments were observed and photographed under a UV transilluminator using a gel documentation system. The banding patterns of the intergeneric F_1_ hybrid and their parents for a specific primer were observed and compared. The reproducibility of the RAPD patterns was confirmed by repeating the PCR analysis [21,22,23]. RAPD markers have been extensively studied and successfully used for the identification and confirmation of F_1_ hybrids in plant breeding [14]. The RAPD analysis is technically simple, cheap, requires little amount of DNA, produces large number of polymorphic markers, and does not require sequence information [15].

### 2.7. Polyploidy Induction

The calli of intergeneric F_1_ hybrids, labelled JR1, JR2, JR3, JR4, JR5, JR6, JR7, and JR8, were obtained in vitro from the young stems and cultured on MS medium enriched with 0.6 mg/L thidiazuron (TDZ), 0.05 mg/L NAA, and 1 mg/L BA for one month at 25 °C with 12 h light (1500 lux). The one-month-old calli were cut into cube sizes of 1 × 1 cm, transferred into a liquid medium of MS supplemented with 0%, 0.3%, 0.4%, and 0.5% colchicine and cultured for three days [5]. Plant polyploidy was identified using manufacturer’s protocols on the flow cytometry (Partec PAII, Sysmex Partec GmbH, Münster, Germany).

### 2.8. Statistical Analysis

The data collected on growth characteristics were subjected to one-way analysis of variance (ANOVA) using SPSS version 16.0 (SPSS Inc., Chicago, IL, USA). Treatment means were compared by Tukey’s test at a 5% significance level. The average values and their corresponding standard deviations were presented.

## 3. Results

### 3.1. Sexual Intergeneric Hybridization and Embryo Rescue 

**Figure 1:**
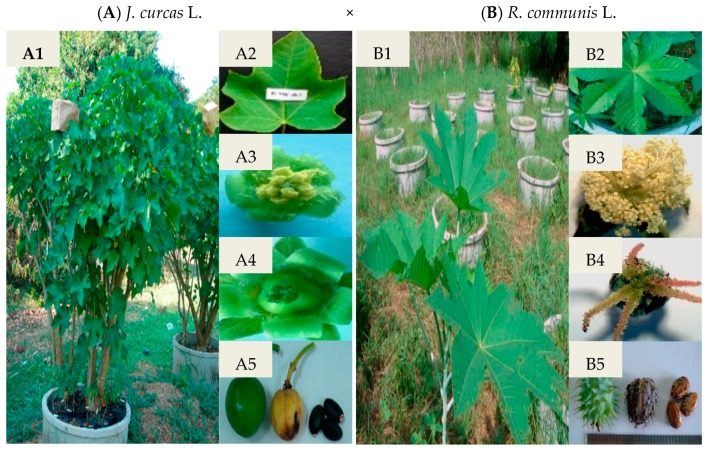
The morphology of *J*. *curcas* L. (**A1**) plant, (**A2**) leaf, (**A3**) male flower, and (**A4**) female flower, (**A5**) fruits and seeds; *R*. *communis* L. (**B1**) plant, (**B2**) leaf, (**B3**) male flower, (**B4**) female flower, and (**B5**) fruits and seeds.

**Figure 2:**
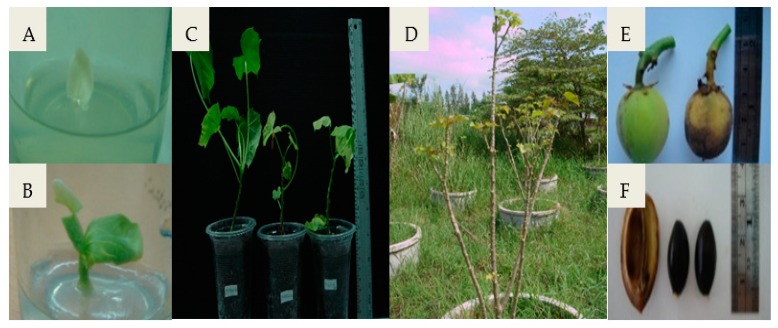
Development of intergeneric F_1_ hybrid from the cross between *J*. *curcas* L. (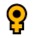) and *R*. *communis* L. (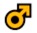), (**A**) embryo rescue, (**B**) three-day-old F_1_ hybrid, (**C**) transfer of F_1_ hybrid to small plastic pots containing sterile peat moss, (**D**) intergeneric hybrid plants in the field, (**E**) fruits, and (**F**) seeds.

**Figure 3:**
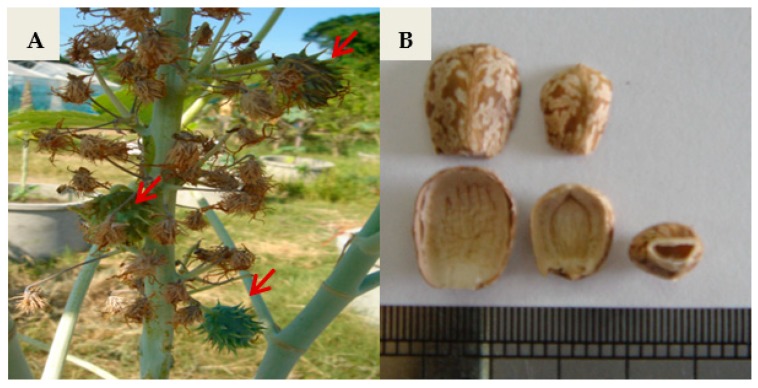
Development of (**A**) fruits and (**B**) seed set of F_1_ hybrid from reciprocal cross between *R*. *communis* (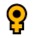) and *J. curcas* (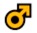).

The effect of GA_3_, NAA, and calcium–boron on fruit set, seed set, and embryo development of the cross and reciprocal cross between the female parent, *J*. *curcas* L., and the male parent, *R*. *communis* L. (Figure 1), were observed and are shown in Table 1. Among the treatments, the intergeneric F_1_ hybrid treated with 0.1% (*v*/*v*) calcium–boron had a high percentage of fruit set (40%) and 100% seedling survival. However, the percentage of seeds set as a result of the application of 25 mg/L GA_3_ and 0.1% (*v*/*v*) calcium–boron on the stigma was the same (83%). The immature embryos rescued by in vitro culture developed into whole plants (Figure 2). Of the 100 intergeneric F_1_ hybrid embryos rescued, 17 regenerated into plantlets, from which eight developed into plants in the field. In contrast, the reciprocal crossing between *J*. *curcas* L. and *R*. *communis* L. was not successful. Embryo abortion was observed 20 days after pollination (Figure 3). The seed capsule was found to be empty with no kernel (Figure 3B).

### 3.2. Identification of Intergeneric F_1_ Hybrid Using Morphological Characteristics

**Figure 4:**
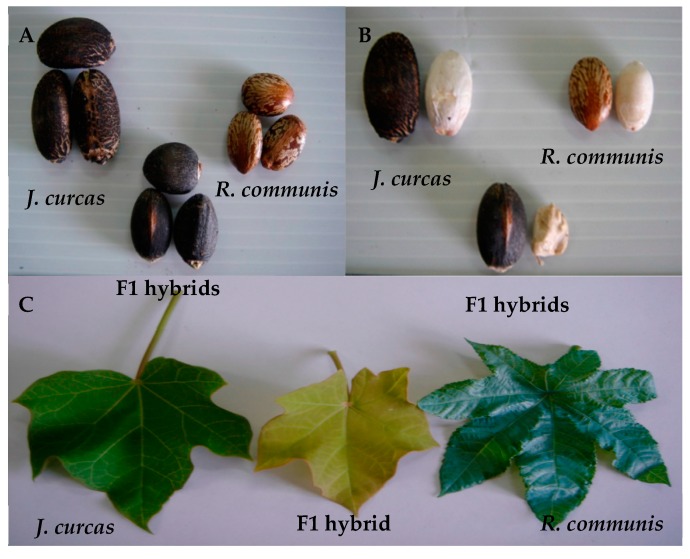
Morphological characteristics of F_1_ hybrid from *J. curcas* (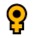) and *R. communis* (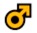) including, (**A**) seeds, (**B**) seed kernel, and (**C**) leaf.

Morphologically, the F_1_ hybrid plants were compared to the parents (Table 2). The results showed that the fruit, seed, and stem of the F_1_ hybrids were similar to those of *J*. *curcas* L. (Figure 1, Figure 2 and Figure 3). The leaf size of the F_1_ hybrid plants was significantly (*p* < 0.05) lower when compared to that of *R*. *communis* L. but similar (*p* > 0.05) to that of *J*. *curcas* L. However, the number of branches per plant and stem diameter of the F_1_ hybrid plants were significantly (*p* < 0.05) higher than those of *R*. *communis* L. but similar (*p* > 0.05) to those of *J*. *curcas* L. The number of female and male flowers of the F_1_ hybrid plants was significantly lower (*p* < 0.05) when compared to that of the parent plants. In addition, the plant height of the F_1_ hybrid plant was significantly (*p* < 0.05) lower when compared to that of *J*. *curcas* L. but similar (*p* > 0.05) to that of *R*. *communis* L. (Table 2). Finally, the seed size of the F_1_ hybrid plants was intermediate between the parent plants (Figure 4). Generally, the F_1_ hybrids exhibited morphological characteristics of both parents.

### 3.3. Identification of Intergeneric F_1_ Hybrid Using RAPD Analysis

**Figure 5:**
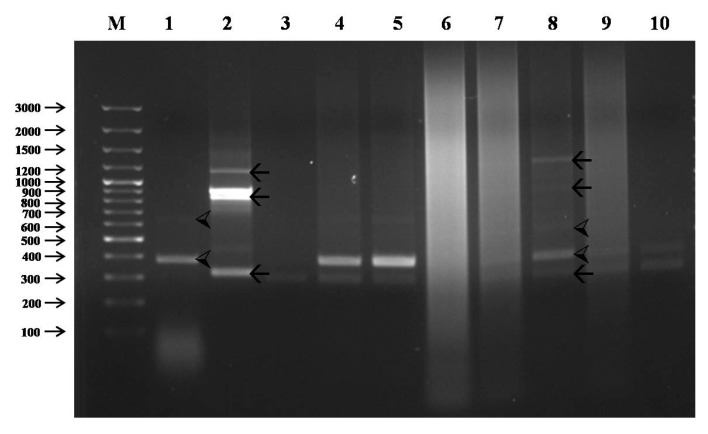
Confirmation of the hybridity of the intergeneric hybrids by randomly amplified polymorphic DNA (RAPD) marker; ⮘—*J*. *curcas* L. specific bands; 🡰—*R*. *communis* L. specific bands; Lane M—ladder; Lane 1—*J*. *curcas* L.; Lane 2—*R*. *communis* L.; Lanes 3–10 are intergeneric hybrids (Lanes 3, 4, 5, 6, 7, 8, 9, and 10 represent JR1, JR2, JR3, JR4, JR5, JR6, JR7, and JR8, respectively). Note: JR represent Jatropha-Ricinus.

RAPD analysis of genomic DNA was performed to confirm the hybridity of the intergeneric F_1_ hybrids. Initially, 45 primers were screened for polymorphism in the parents. From these 45, only 10 distinguished the parents. The 10 primers (OPA-01–OPA-10) were used to amplify DNA from the F_1_ hybrids along with the parents. Out of the 10 primers, only one (OPA-07) generated polymorphic PCR bands of both parents in the F_1_ hybrids of *J*. *curcas* L. and *R*. *communis* L. (Figure 5). The clear and specific amplified DNA fragments of *J*. *curcas* L. were 400 and 600 bp, whereas those of *R*. *communis* L. were 300, 400, 800, 900, and 1200 bp. Similarly, the hybrids showed specific bands of *J*. *curcas* L. and *R*. *communis* L. The F_1_ hybrid JR6 showed the clearest specific bands of both *J*. *curcas* L. (400 and 600 bp) and *R*. *communis* L. (300, 400, 800, 900, and 1200 bp) (Figure 5).

### 3.4. Induction of Polyploids in the Intergeneric Hybrids

**Figure 6:**
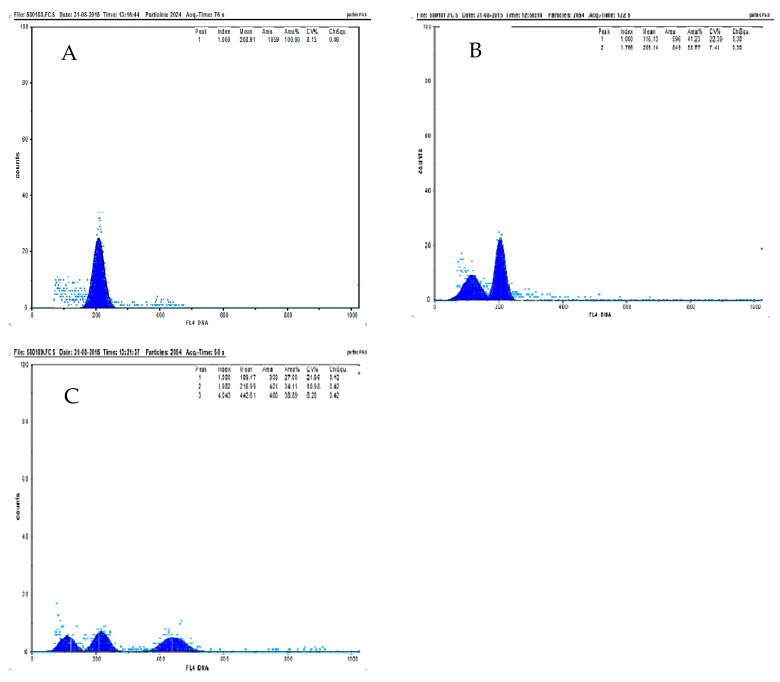
Histogram of the relative fluorescence intensity of nuclei isolated from (**A**) *J*. *curcas* L., (**B**) *R*. *communis* L., and (**C**) intergeneric hybrid (JR6) treated with 0.3% colchicine for three days.

The calli JR1 and JR6 successfully responded to the colchicine application in vitro (Table 3). The ploidy percentage was determined from the peak area of the histogram of the relative fluorescence intensity of nuclei of each plant using flow cytometry. The diploid *J*. *curcas* L. showed one clear peak at channel 200 (Figure 6A) while *R*. *communis* L. presented peaks at channel 100 and 200 (Figure 6B). The highest tetraploid percentage (38.89%) was observed in the F_1_ hybrid JR6 after treatment with 0.3% colchicine for three days (Figure 6C).

## 4. Discussion

Intergeneric hybridization is a very useful technique for improving crop species worldwide [16]. It has been used to develop several F_1_ hybrid plants with genetic variability from the parent plants [16,24,25]. However, one major barrier to intergeneric hybridization is the problem of very low or no seed set [26]. Tang [25], for example, reported the absence of seeds in F_1_ hybrids after an intergeneric hybridization between *Dendranthema nankingense* and *Tanacetum vulgare*. Zhao [24] also reported only 5% seed set after an intergeneric cross between *Dendranthema* × *grandiflorum* “Aoyunhuoju” and *Ajania pacifica*. To overcome this barrier, several chemical formulations have been used by different researches to enhance seed set. Dinesh [27] reported 13.1% seed set per fruit after application of 5% sucrose to the stigma of *Vasconcellea cauliflora* during the intergeneric crossing between *Carica papaya* var. Surya and *V. cauliflora*. Jayavalli [28] also reported an enhancement in both fruit and seed set after application of 5% sucrose, 0.5% boron + 5% sucrose, and 0.5% CaCl_2_ + 5% sucrose during the intergeneric hybridization of *C. papaya* and *V. cauliflora*. Application of 0.1% calcium–boron to the stigma of the female parent plant (*J*. *curcas* L.) in the current study resulted in 100% intergeneric F_1_ hybrid seedling survival with high fruit set and seed set (Table 1). Treating the stigma of the female parent plant with GA_3_, NAA, and calcium–boron may promote early pollen germination, increase the period of pollination, and improve the growth of the pollen tube because these compounds influence fertilization and increase cell division during fertilization [29].

The F_1_ hybrid seeds in this study however were shrunken with slimmed embryos (Figure 1), an observation that has been reported in other research [14,30]. Interspecific or intergeneric hybridization is limited by postzygotic incompatibilities including embryo abortion and degeneration, resulting in a decrease in fertility. However, postzygotic incompatibilities can be overcome by the use of embryo rescue techniques [31]. Embryo rescue has been successfully used to overcome postzygotic incompatibilities following interspecific or intergeneric hybridization of a wide range of plant species, subsequently leading to the development F_1_ hybrids [14,30,32]. An average regeneration efficiency of only 17% was recorded after the embryo rescue of F_1_ hybrid embryos in the current study, which may be due to several factors including the media composition and the developmental stage of the embryo [31]. Rodrangboon [14] reported an average in vitro embryo regeneration efficiency of 25.6% for F_1_ hybrid (*Oryza sativa* × *Oryza officinalis*) embryos obtained 7–10 days after pollination. The efficiency however increased to 55.6% for embryos obtained 11–14 days after pollination. In vitro culturing of F_1_ hybrid (*Brachiaria ruziziensis* × *Brachiaria decumbens* or *Brachiaria brizantha*) of 9–12 days old embryos yielded over 80% regeneration efficiency unlike approximately 35% for 7–8 days old embryos [30]. Excising the F_1_ hybrid embryos at the optimal developmental stage and adjusting the composition of the RM as well as the culture conditions in the current study may enhance the efficiency of regeneration [31].

The F_1_ hybrid plants showed morphological resemblance to both parents, a clear indication that they are from the intergeneric crossing, however, hybridity was confirmed using a RAPD marker. RAPD analysis is one of the most commonly used molecular techniques for the study of genetic diversity [21]. It has been successfully used to confirm hybridity in F_1_ hybrids including *Passiflora* [11], *Solanum* [33], *Capsicum* [34], and *Centaurium* [13]. The clear expression of the specific bands of both parents in the F_1_ hybrid JR6 (Figure 5) is a confirmation of hybridity and the authenticity of this particular hybrid plant in the current study. This result confirms the successful production of an intergeneric F_1_ hybrid from the female parent, *J*. *curcas* L., and the male parent, *R*. *communis* L.

Sterility of F_1_ hybrids has often been observed in intergeneric hybridization [14]. Polyploidization in F_1_ hybrids is a process that can help to overcome sterility. Polyploids can be induced in the F_1_ hybrids through the application of various antimitotic agents, including colchicine, trifluralin, oryzalin, and amiprophos-methyl [6]. However, polyploidy induction has mainly been accomplished by the use of colchicine. Induction of polyploids through the application of colchicine has been reported by several researchers [35,36,37]. In the current study, attempts were made to double the chromosome set of the F_1_ hybrid through the application of colchicine. However, the tetraploidy percentage was low. The concentration and duration of colchicine application, among other factors, plays a key role in the success of polyploidy induction [38]. Polyploidy has been successfully induced in *Lychnis senno* with the application of colchicine concentrations as low as 0.00001% [35] and in *Chaenomeles japonica* with a very high concentration of 1.5% [39]. Several studies have shown that the application of high colchicine concentrations for lengthy periods is effective in the successful induction of polyploids [36,40,41]. However, very high colchicine concentrations for a longer time have been reported by several researches to be very harmful to the explants [35,38,39]. Expanding the range of colchicine concentrations and varying its application period, with a balance between survival and successful polyploidy induction in mind, may help to improve the polyploidization process in the current study. Castro [37] recommended the selection of seedlings at the ideal stage of development for the polyploidization process after reporting the efficient induction of polyploidy in younger seedlings when compared to older ones. Unfortunately, this factor has not been well exploited by most researchers. The calli age and stage of development may also be exploited to enhance the polyploidization process in the current study.

## 5. Conclusions

An intergeneric F_1_ hybrid from the female parent, *J. curcas* L., and the male parent, *R. communis* L., was produced. The hybridity and authenticity of the F_1_ hybrid was successfully confirmed. However, a low tetraploidy percentage was recorded. Expanding the range of colchicine concentrations and varying its application period as well as exploiting the calli age and stage of development may help to enhance the polyploidization process. The results in the current study form a strong basis for further research to enhance intergeneric hybrid production from *J*. *curcas* L. and *R. communis* L.

## Figures and Tables

**Table 1:** Effect of GA_3_, NAA, and calcium–boron on sexual intergeneric hybridization.

Cross	Chemicals	Fruit Set (%)	Seed Set (%)	Seed Length (mm)	Seed Width (mm)	Seed Weight (g)	Seedling Survival (%)
JC	Control	100	93	18.79 ± 0.16	11.26 ± 0.07	1.14 ± 0.10	100
JC × RC	GA_3_ 25 mg/L	15	83	25.07 ± 1.00	28.13 ± 0.82	1.00 ± 0.07	10
	GA_3_ 50 mg/L	20	83	27.59 ± 0.31	30.37 ± 0.80	1.04 ± 0.06	80
	NAA 25 mg/L	12.5	66	27.13 ± 1.37	24.06 ± 1.00	1.10 ± 0.00	0
	NAA 50 mg/L	0	-	-	-	-	-
	Calcium–boron 0.1% (*v*/*v*)	40	83	25.00 ± 2.09	27.28 ± 0.62	0.94 ± 0.06	100
	Calcium–boron 0.2% (*v*/*v*)	0	-	-	-	-	-
RC	Control	76	4	16.41 ± 0.50	18.36 ± 0.51	0.20 ± 0.09	63
RC × JC	GA_3_ 25 mg/L	4	56	8.88 ± 0.72	6.01 ± 0.15	0.03 ± 0.01	EB
	GA_3_ 50 mg/L	0	-	-	-	-	-
	NAA 25 mg/L	3	66	9.34 ± 1.42	6.24 ± 0.85	0.04 ± 0.01	EB
	NAA 50 mg/L	0	-	-	-	-	-
	Calcium–boron 0.1% (*v*/*v*)	8	24	13.70 ± 0.61	12.84 ± 1.49	0.12 ± 0.06	EB
	Calcium–boron 0.2% (*v*/*v*)	31	9	13.37 ± 0.17	12.35 ± 0.51	0.24 ± 0.03	EB

JC represents *Jatropha* curcas L.; RC represents *Ricinus communis* L.; EB represents embryo abortion.

**Table 2:** Growth characteristics of the intergeneric hybrids.

Characters	*J. curcas* L.	*R. communis* L.	Intergeneric Hybrids
Plant height (mm)	2070.33 ± 11.14 ^a^	1630.00 ± 6.54 ^b^	1620.00 ± 4.54 ^b^
No. of branches per plant	4.00 ± 0.28 ^a^	3.00 ± 0.19 ^b^	4.67 ± 0.38 ^a^
No. of female flowers	9.00 ± 0.53 ^b^	34.00 ± 0.98 ^a^	0.50 ± 0.05 ^c^
No. of male flowers	144.00 ± 5.25 ^a^	128.50 ± 4.55 ^b^	30.00 ± 1.24 ^c^
Stem diameter (mm)	280.67 ± 0.50 ^a^	90.00 ± 0.35 ^b^	280.33 ± 0.53 ^a^
Leaf size length (mm)	100.17 ± 0.47 ^b^	190.30 ± 0.69 ^a^	98.80 ± 0.41 ^b^
Leaf size width (mm)	110.43 ± 0.68 ^b^	230.00 ± 0.45 ^a^	110.67 ± 0.76 ^b^

Data are presented as mean ± SD. Means with similar superscript (^a,b,c^) in the same row are not significantly different (*p* > 0.05).

**Table 3:** Ploidy level of callus treated with colchicine.

Callus	Colchicine (%)	Treatment Time (Days)	Peak 1 (% Area)	Peak 2 (% Area)	Peak 3 (% Area)
JR1	0	3	100	-	-
	0	7	18.64	71.10	10.25
	0.3	3	26.91	44.20	28.89
	0.3	7	39.40	38.48	22.12
	0.4	3	32.24	47.77	20.00
	0.5	3	38.12	44.20	17.68
JR6	0	3	100	-	-
	0.3	3	27.00	34.11	38.89
	0.4	3	59.37	24.12	16.51
	0.5 *	3	-	-	-

Note: * represents contamination.
